# Integrated approaches for pathogen monitoring and shotgun metagenomic analysis in Atlantic salmon farming

**DOI:** 10.1038/s41598-026-48791-x

**Published:** 2026-04-14

**Authors:** Ottavia Benedicenti, David A. Strand, Saima Nasrin Mohammad, Snorre Gulla, Marit Måsøy Amundsen, Hilde Sindre, Trude Vrålstad

**Affiliations:** Norwegian Veterinary Institute, Postboks 64, 1431 Ås, Norway

**Keywords:** Atlantic salmon pathogens, Environmental DNA, Non-invasive sampling, Shotgun metagenomic sequencing, Biological techniques, Biotechnology, Environmental sciences, Microbiology

## Abstract

Specific tools for detecting waterborne pathogens are essential for limiting disease spread in aquaculture. We evaluated a field-deployable workflow combining filtration of eDNA/eRNA with targeted (RT-)qPCR and complementary shotgun metagenomics to monitor pathogens and microbial community dynamics in a single-farm study following one Atlantic salmon production cohort from hatchery to slaughter. The primary aim was to assess workflow feasibility and performance under real farm conditions, while secondarily examining whether metagenomic profiles could contextualise microbial shifts associated with pathogen presence. ISAV was consistently detected in hatchery water at ~ 4 × 10^3^–9 × 10^3^ copies/L, whereas PRV1 was detected only inside sea pens from August onward (~ 4 × 10^2^–1.5 × 10^4^ copies/L) and increased by more than two orders of magnitude after wellboat delousing. Shotgun metagenomics yielded a median of ~ 1.5 × 10^5^ reads per sample (mean read length ~ 2.5 kb; N50 > 2 kb), enabling broad taxonomic screening. PRV1-positive seawater samples showed modest decreases in richness and shifts in viral taxa, though patterns were subtle and should be interpreted cautiously given low pathogen loads. The workflow was practical for trained farm personnel, and this integrated approach offers a scalable system for routine pathogen surveillance and supports earlier, evidence-based biosecurity actions, providing broader microbial information than qPCR alone.

**Supplementary Information:**

The online version contains supplementary material available at 10.1038/s41598-026-48791-x.

## Introduction

The production of Atlantic salmon, *Salmo salar* (Linnaeus), reached approximately 2.02 million tonnes during January–September 2024, representing a slight 1% decline compared to 2023, with Norway remaining the main producer (ca. 1.5 million tonnes; − 1.3% year-on-year)^1^. Despite the scale and economic importance of this industry, infectious diseases continue to pose major challenges, negatively affecting fish health, welfare, and productivity^2^. Effective pathogen surveillance is therefore essential to support timely mitigation strategies and to limit pathogen spread within and between aquaculture facilities.

Traditional health monitoring in aquaculture relies largely on direct fish sampling, which can be invasive, labour-intensive, and limited in its ability to capture early or subclinical infections. In this context, environmental DNA/RNA (eDNA/eRNA) sampling has emerged as a promising alternative for pathogen surveillance^3–9^. By analysing water samples, this approach enables the detection of pathogens shed by infected fish as well as those circulating within the farming environment. Such non-invasive monitoring may allow earlier detection of infection events, supporting proactive management decisions before clinical signs become evident. Furthermore, water-based surveillance aligns with 3R (Replacement, Reduction, Refinement) principles^10^ and the One Health framework, promoting integrated approaches that benefit animal welfare, environmental sustainability, and human health^11^.

High-throughput sequencing approaches, including metabarcoding and shotgun metagenomics, have increasingly been applied to characterise microbial communities in aquaculture systems, particularly in recirculating aquaculture systems (RAS), where microbial processes are central to nutrient cycling, water quality, and fish health^12–14^. Different sequencing strategies provide complementary insights: for example, 16S rRNA gene sequencing is effective for describing community structure, while long-read sequencing and shotgun metagenomics can offer improved taxonomic resolution and enable detection of a broader range of organisms, including viruses and fungi^12,14,15^. However, methodological choices, including sequencing platforms, analytical pipelines, and reference databases, can strongly influence results and may limit comparability across studies. Indeed, a substantial proportion of environmental microbial diversity, particularly viral diversity, remains uncharacterised^16^. While metagenomics has shown promise as a non-invasive tool for environmental monitoring and disease prevention^13^, its application in tank- and cage-based salmon farming systems remains relatively limited, and its practical value for routine pathogen surveillance under field conditions is still not fully established^17^.

Given these limitations, there is a need for practical and scalable surveillance approaches that combine sensitive pathogen detection with broader ecological context, while being applicable under real farming conditions. In this context, integrating targeted molecular assays with metagenomic profiling may improve detection capabilities and provide additional insight into environmental changes associated with pathogen presence. Therefore, this study explored a non-invasive workflow for monitoring pathogens in aquaculture environments based on a previously established method for filtering, storing, and extracting eDNA/eRNA from water samples^18^.

Specifically, we applied a combined approach integrating targeted qPCR detection with shotgun metagenomic profiling to assess pathogen presence and microbial community dynamics in both hatchery and seawater sites, following the same Atlantic salmon population. Pathogen surveillance during operational stress events, such as wellboat delousing, revealed increased pathogen concentrations in water samples following treatment. In addition, we evaluated the feasibility of on-site microbial screening using portable sequencing technologies (Oxford Nanopore MinION), enabling rapid field-based analysis. Preliminary metagenomic results provided insights into microbial community shifts, including changes in richness and virome composition, potentially associated with pathogen presence and environmental disturbance. To improve taxonomic resolution, custom pathogen databases were also used alongside publicly available resources, highlighting the importance of curated reference data for accurate metagenomic interpretation.

The overall aim of this study was to evaluate the feasibility and performance of a field-deployable workflow for pathogen surveillance in aquaculture, combining on-site water sampling, targeted qPCR detection, and complementary metagenomic analysis. In addition to assessing methodological performance under real farm conditions, we investigated whether shotgun metagenomics could provide added ecological context relevant to pathogen occurrence, thereby supporting more comprehensive health-monitoring frameworks in aquaculture.

## Results

### qPCR / RT-qPCR assays

The same Atlantic salmon population was monitored from the hatchery stage (freshwater and smoltification process) through transfer to the seawater site. Across all sampling points, qPCR/RT-qPCR detected two target pathogens: infectious salmon anaemia virus (ISAV) in hatchery inlet, tank, and outlet waters, and piscine orthoreovirus genotype 1 (PRV1) in seawater cage samples from August 2023 onwards (Tables 1 and S2). No pathogens were detected in offshore samples (1 km). PRV1 was consistently detected only inside the pens, except during the final sampling event, when low-level PRV1 signals were also observed 200 m from the cage. Additional wellboat sampling before and after delousing showed increased PRV1 concentrations after treatment, consistent with enhanced viral shedding during handling stress (Tables 1 and S2).

**Table 1:** Cq values and RT-qPCR assays estimated copy numbers per L for ISAV (hatchery) and PRV1 (seawater sites and wellboat). Results are shown as mean ± standard deviation from three biological replicates and two technical replicates.

Samples	Group	Cq mean ± St. Dev.	Copy numbers/L ± St. Dev.
Outlet water (Feb/23)	Hatchery	35.39 ± 0.37	3952.00 ± 1012.58
Tank water (Feb/23)	Hatchery	35.42 ± 0.09	3824.20 ± 254.28
Inlet water (Feb/23)	Hatchery	34.68 ± 0.23	6414.00 ± 1009.75
Control (Feb/23)	Hatchery	N/A	N/A
Outlet water (May/23)	Hatchery	34.30 ± 0.08	8370.00 ± 444.06
Tank water (May/23)	Hatchery	34.25 ± 0.30	8700.00 ± 1753.62
Inlet water (May/23)	Hatchery	34.38 ± 0.12	7884.00 ± 661.85
Control (May/23)	Hatchery	N/A	N/A
1 km from cage − 1 m depth (Apr/23)	Seawater sites	N/A	N/A
1 km from cage − 20 m depth (Apr/23)	Seawater sites	N/A	N/A
Seawater cage (Apr/23)	Seawater sites	N/A	N/A
Control (Apr/23)	Seawater sites	N/A	N/A
1 km from cage − 1 m depth (May/23)	Seawater sites	N/A	N/A
1 km from cage − 20 m depth (May/23)	Seawater sites	N/A	N/A
Seawater cage (May/23)	Seawater sites	N/A	N/A
Control (May/23)	Seawater sites	N/A	N/A
Seawater cage (Jun/23)	Seawater sites	N/A	N/A
Seawater cage (Jul/23)	Seawater sites	N/A	N/A
Seawater cage (Aug/23)	Seawater sites	38.00 ± 0.66	209.94 ± 91.78
Control (Aug/23)	Seawater sites	N/A	N/A
Seawater cage (Sep/23)	Seawater sites	31.68 ± 0.06	14826.00 ± 557.20
1 km from cage − 1 m depth (Oct/23)	Seawater sites	N/A	N/A
1 km from cage − 20 m depth (Oct/23)	Seawater sites	N/A	N/A
Seawater cage (Oct/23)	Seawater sites	34.12 ± 0.08	2800.80 ± 162.92
Control (Oct/23)	Seawater sites	N/A	N/A
Wellboat before treatment (Nov/23)	Wellboat	36.81 ± 0.77	481.78 ± 242.99
Wellboat after treatment (Nov/23)	Wellboat	29.93 ± 0.07	48760.00 ± 2319.31
Seawater cage (Dec/23)	Seawater sites	32.15 ± 0.02	10770.00 ± 132.94
1 km from cage − 1 m depth (Feb/24)	Seawater sites	N/A	N/A
1 km from cage − 20 m depth (Feb/24)	Seawater sites	N/A	N/A
Seawater cage (Feb/24)	Seawater sites	39.20 ± 1.46	111.30 ± 95.69
Control (Feb/24)	Seawater sites	N/A	N/A
200 m from cage − 1 m depth (Feb/24)	Seawater sites	40.09 ± 0.01	47.80 ± 0.45
200 m from cage − 20 m depth (Feb/24)	Seawater sites	N/A	N/A

Although some detections occurred at high Cq values (> 35), these were consistent with low-concentration environmental samples near the assay limit of detection (LOD). For these samples, copy number estimates are subject to increased stochastic variability and should therefore be interpreted qualitatively (detected vs. not detected) rather than quantitatively. Only detections with Cq values below this range were considered sufficiently reliable for quantitative comparison. Importantly, low-positive results were reproducible across biological (*n* = 3) and technical (*n* = 2) replicates and supported by standard curve–based quantification (Table S2), indicating true low-level environmental shedding rather than analytical noise.

### Sequencing pipeline output and quality assessment

Oxford Nanopore ligation data were processed with the NEPAL pipeline using three strategies: simplex, duplex, and nanofilt-filtered reads. Five key metrics were analysed for each run (runs 1–4) using boxplots (Fig. S1 and Table S3): (1) average read length, which varied between runs, with duplex reads generally shorter due to trimming during consensus generation; (2) average quality scores (range: Q15–Q21), which differed significantly among workflows, with duplex reads exhibited the highest average quality, while nanofilt results depended on the filtering threshold; (3) GC content (%), which remained relatively stable across workflows and runs (35–50%, as expected for environmental samples); (4) N50 (bp) values used as an indicator of assembly performance, with values exceeding 2000 bp; and (5) Q30 (%), where duplex reads showed lower proportions of high-confidence bases compared to nanofilt-filtered reads. Based on these results, we proceeded with nanofilt-filtered reads for taxonomic profiling using the taxprofiler pipeline with the Kraken2 database. Although filtering may remove some informative reads, Kraken2 benefits from longer reads and higher Q30 values; therefore, nanofilt-filtered reads were considered more robust for taxonomic classification, offering improved sensitivity and specificity. The taxprofiler output was assessed using standard quality metrics (Fig. S2 and Table S4). As expected, control samples exhibited a lower number of reads compared to experimental samples (Fig. S2 and Table S4). Median read lengths ranged from 1749 to 2749 bp, and GC content was consistent across samples (≈ 40–50%), supporting overall library uniformity (Fig. S2 and Table S4). However, *Homo sapiens* reads were detected in several samples, suggesting low-level contamination introduced during field sampling rather than library preparation (Fig. S2 and Table S4).

### Taxonomic classification

We first analysed the overall relative abundance (%) among the top six classified phyla, excluding the *S. salar* genome (taxprofiler pipeline) and *H. sapiens* contamination. Samples were grouped by environment (hatchery, seawater sites, and wellboat) and controls (Fig. S3). Hatchery samples exhibited the lowest proportion of unclassified reads (12.82 ± 6.37%) and minimal variability in “other” taxa (1.88 ± 0.07%) (Fig. S3 and Table S5). In contrast, seawater sites showed higher unclassified percentages (23.99 ± 4.71%) and greater variability in “other” taxa (5.03 ± 1.25%) (Fig. S3 and Table S5). The wellboat samples were the most variable, largely due to the post-treatment sample, which showed 43.5% unclassified reads (Fig. S3 and Table S5). Such increases are common in environmental metagenomics when communities contain poorly represented taxa or sequences requiring de novo assembly for classification^19^. The Zymo microbial standard positive control was almost fully classified (99.99%), with negligible unclassified or “other” taxa (0.01%) (Fig. S3 and Table S5), as expected for a well-characterised mock community. Similarly, the positive control from the spiked pilot experiment^18^ was also nearly fully classified (97.56%), with only a small fraction unclassified (1.53%) (Fig. S3 and Table S5). These results confirm that the pipeline performed well on known or control samples.

**Fig. 1:**
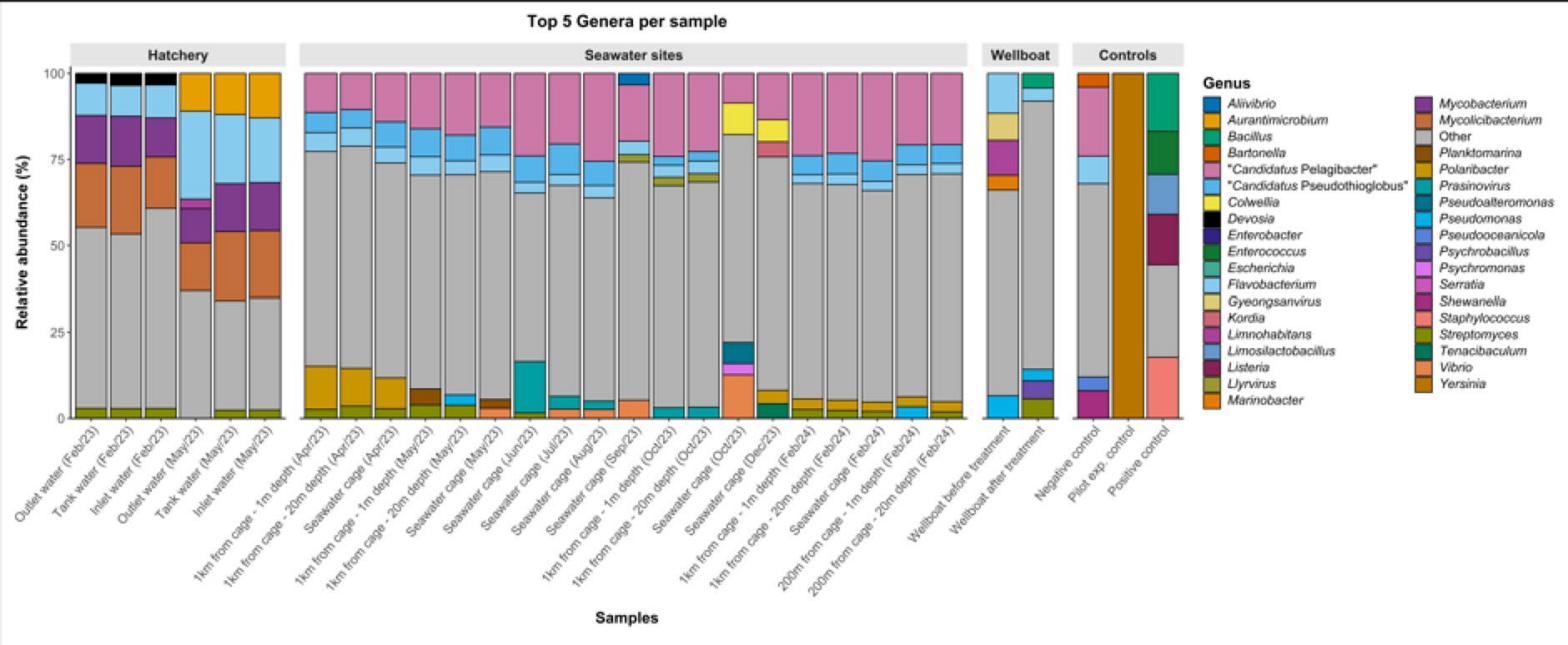
Relative abundance (%) of the top five genera per sample after nanofilt processing. Samples are grouped by origin: hatchery, seawater sites, wellboat, and controls. Genera are colour-coded, and “other” represents taxa outside the top five for each sample.

A general taxonomic composition profile showed a shift from *Actinomycetota* (phylum of Gram-positive bacteria with high GC content) and *Bacteroidota* (Gram-negative bacteria) dominance in the hatchery, to more diverse communities in seawater sites reflecting environmental changes (Fig. S3). Hatchery water is nutrient-rich and controlled, favouring the enrichment of heterotrophic degraders typical of recirculating systems^20,21^, while seawater sites showed: *Chlorophyta* peaks during spring/algal bloom periods, indicating seasonal eutrophication and/or phytoplankton proliferation; *Nitrososphaerota* presence, which suggest active nitrification, likely linked to nitrogen cycling in the marine environment^22^; and *Uroviricota* and *Nucleocytoviricota*, which could be correlated to viral regulation of microbial and algal populations, influencing bloom dynamics and nutrient turnover^23,24^ (Fig. S3). Because Eukaryota and Archaea were particularly abundant in marine samples and could mask bacterial and viral patterns, subsequent analyses focused on prokaryotic and viral taxa. Therefore, we showed the relative abundance (%) of the top five genera per sample after nanofilt processing, including “others” (Fig. 1 and Table S6). Hatchery samples showed the highest percentage for “other” (ca. 22.08%), but relatively low unclassified reads, while seawater sites had higher unclassified reads (ca. 23.99%), reflecting marine complexity with taxonomic gaps (Table S7). The extreme unclassified proportion (43.50%) was driven by the wellboat post-treatment sample, while controls behaved as expected with low unclassified reads except for minor reagent contamination (negative control) (Table S7). Hatchery microbiota was dominated by *Mycolicibacterium* (*Actinomycetota*), *Mycobacterium* (*Actinomycetota*) and *Flavobacterium* (*Bacteroidota*) (Fig. 1 and Table S6). In contrast, seawater sites exhibited seasonal shifts that strongly influenced microbial composition: spring blooms (April–May) favoured polysaccharide degraders (*Polaribacter*^25)^, along with oligotrophic specialists such as “*Candidatus* Pelagibacter” (SAR11^26^), summer (July–August) was characterised by viral–algal interactions (*Prasinovirus*^27)^, autumn (September–October) introduced potential opportunistic pathogens (*Aliivibrio*^28^, *Pseudalteromonas*^29)^, and winter (December–February) was dominated by “*Candidatus* Pelagibacter” (SAR11), together with cold-adapted genera like *Colwellia*^30^ and bloom-termination-associated genera such as *Kordia*^31^. Negative controls contained a high proportion of “other” genera (59.94%), consistent with low-level reagent contamination typical of low-biomass samples. The pilot positive control experiment^18^ was dominated by *Yersinia* (99.99%), and the Zymo microbial standard showed the expected balanced composition, further supporting sequencing and classification accuracy^32^ (Fig. 1 and Table S6).

### Alpha and beta diversities

**Fig. 2:**
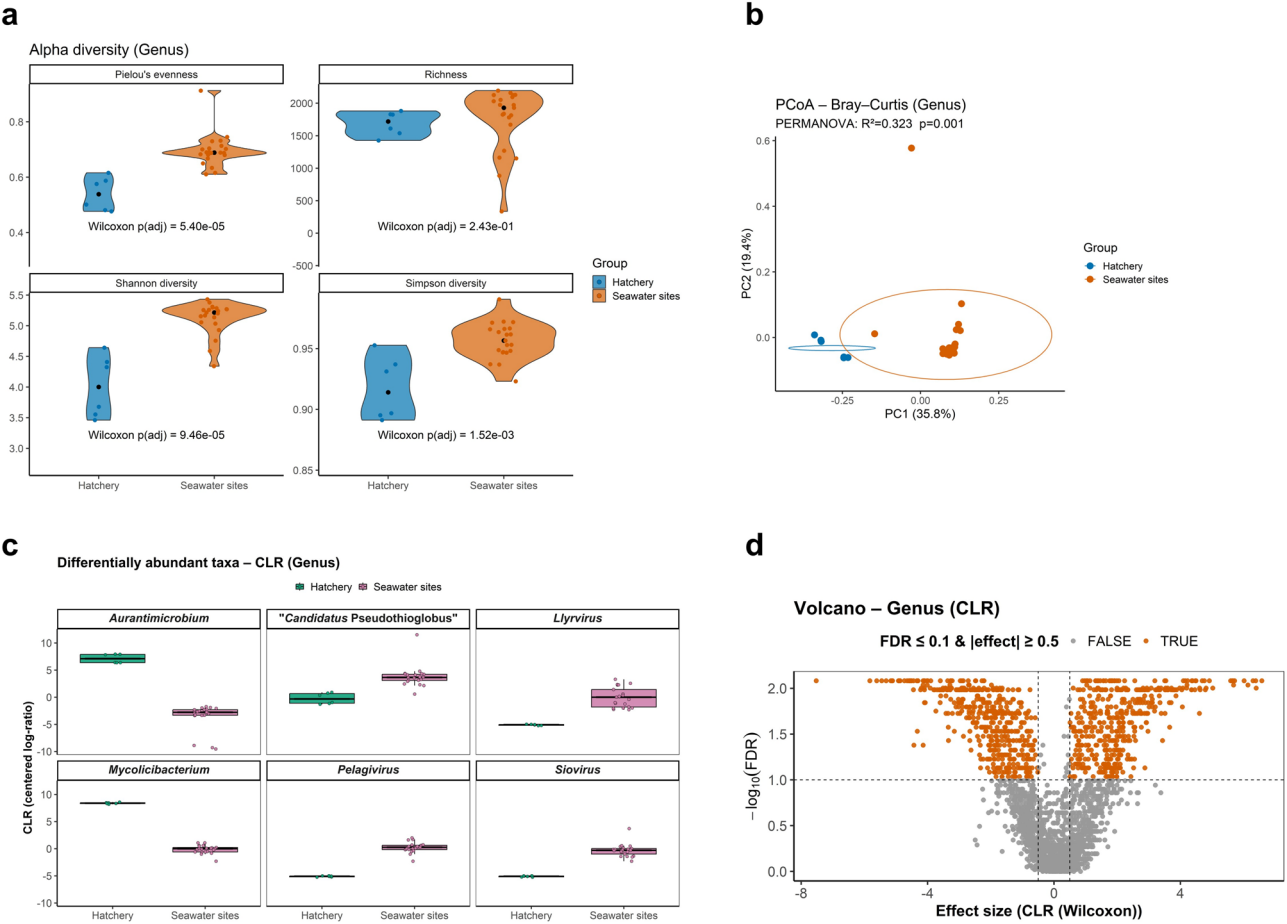
Alpha and beta diversity analyses and differentially abundant taxa at the genus level after excluding *Homo sapiens*, Archaea, and Eukaryota. (**a**) Alpha diversity metrics (Pilou’s evenness, richness, Shannon diversity, and Simpson diversity) with a Wilcoxon significant test. (**b**) Beta diversity based on Bray–Curtis dissimilarity indicates clear separation between hatchery and seawater samples (PERMANOVA R^2^ = 0.323, *p* = 0.001). (**c**) Differential abundance analysis of the top five genera using centred log-ratio (CLR) transformation highlights genera enriched in hatchery versus seawater sites. (**d**) Volcano plot of CLR effect sizes (Wilcoxon test) and FDR-adjusted p-values identifies taxa with significant differences (FDR ≤ 0.1 and |effect size| ≥ 0.5).

Alpha and beta diversity analyses between hatchery and seawater sites were not significantly different at the phylum or species level; therefore, subsequent analyses focused on the genus level, as this was considered the lowest phylogenetic rank providing reliable information based on the selected standard Kraken2 database. At this level, alpha diversity differed significantly in Pielou’s evenness, Shannon, and Simpson indices, while richness did not differ significantly between environments (Wilcoxon test; Fig. 2a). Beta diversity based on Bray–Curtis dissimilarity^33^ showed clear separation between hatchery and seawater samples (PERMANOVA, R^2^ = 0.323, *p* = 0.001; Fig. 2b). Differential abundance analysis of the six most abundant genera using centred log-ratio (CLR) transformation identified consistent compositional differences between environments. Hatchery showed higher relative abundance in *Aurantimicrobium* and *Mycolicibacterium*, while marine sites in “*Candidatus* Pseudothioglobus”, *Lyrvirus*, *Pelagivirus*, and *Siovirus* (Fig. 2c). Volcano plots of CLR effect sizes (Wilcoxon test^34^ and false discovery rate (FDR)-adjusted p-values^35^ indicated a consistent set of genera significantly different between environments (FDR ≤ 0.1 and |effect size| ≥ 0.5; Fig. 2d). Effect sizes (CLR) are reported alongside FDR-adjusted p-values in order to highlight not only statistically significant differences, but also the magnitude of compositional shifts between environments. The use of an effect size threshold (|effect size| ≥ 0.5) ensures that only taxa showing biologically meaningful changes are retained for interpretation.

**Fig. 3:**
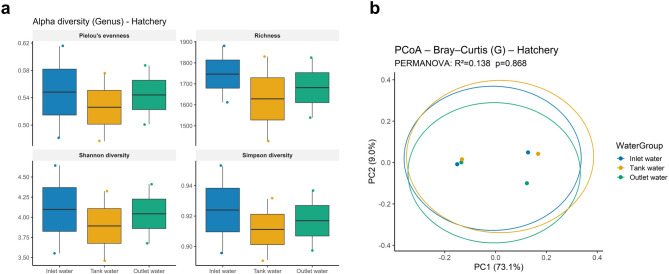
Alpha and beta diversity of hatchery water samples at the genus level after excluding *Homo sapiens*, Archaea, and Eukaryota. (**a**) Boxplots show alpha diversity metrics (Pielou’s evenness, richness, Shannon, and Simpson) across three water groups: inlet, tank, and outlet water. No significant differences were observed among these groups for any metric. (**b**) Principal Coordinates Analysis (PCoA) based on Bray–Curtis dissimilarity illustrates beta diversity patterns among the same water groups. PERMANOVA results (R^2^ = 0.138, *p* = 0.868) indicate no significant compositional differences between inlet, tank, and outlet water samples.

**Fig. 4:**
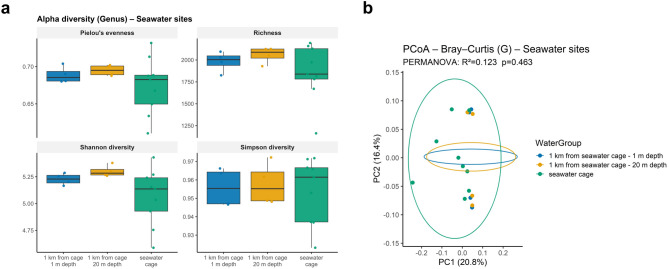
Alpha and beta diversity of seawater samples at the genus level after excluding *Homo sapiens*, Archaea, and Eukaryota. (**a**) Boxplots show alpha diversity metrics (Pielou’s evenness, richness, Shannon, and Simpson) across three sampling conditions: 1 km from the seawater cage at 1 m depth, 1 km from the cage at 20 m depth, and directly at the seawater cage. No significant differences were observed among these groups for any metric. (**b**) Principal Coordinates Analysis (PCoA) based on Bray–Curtis dissimilarity illustrates beta diversity patterns among the same water groups. PERMANOVA results (R^2^ = 0.123, *p* = 0.463) indicate no significant compositional differences between sampling depths or proximity to the cage.

**Fig. 5:**
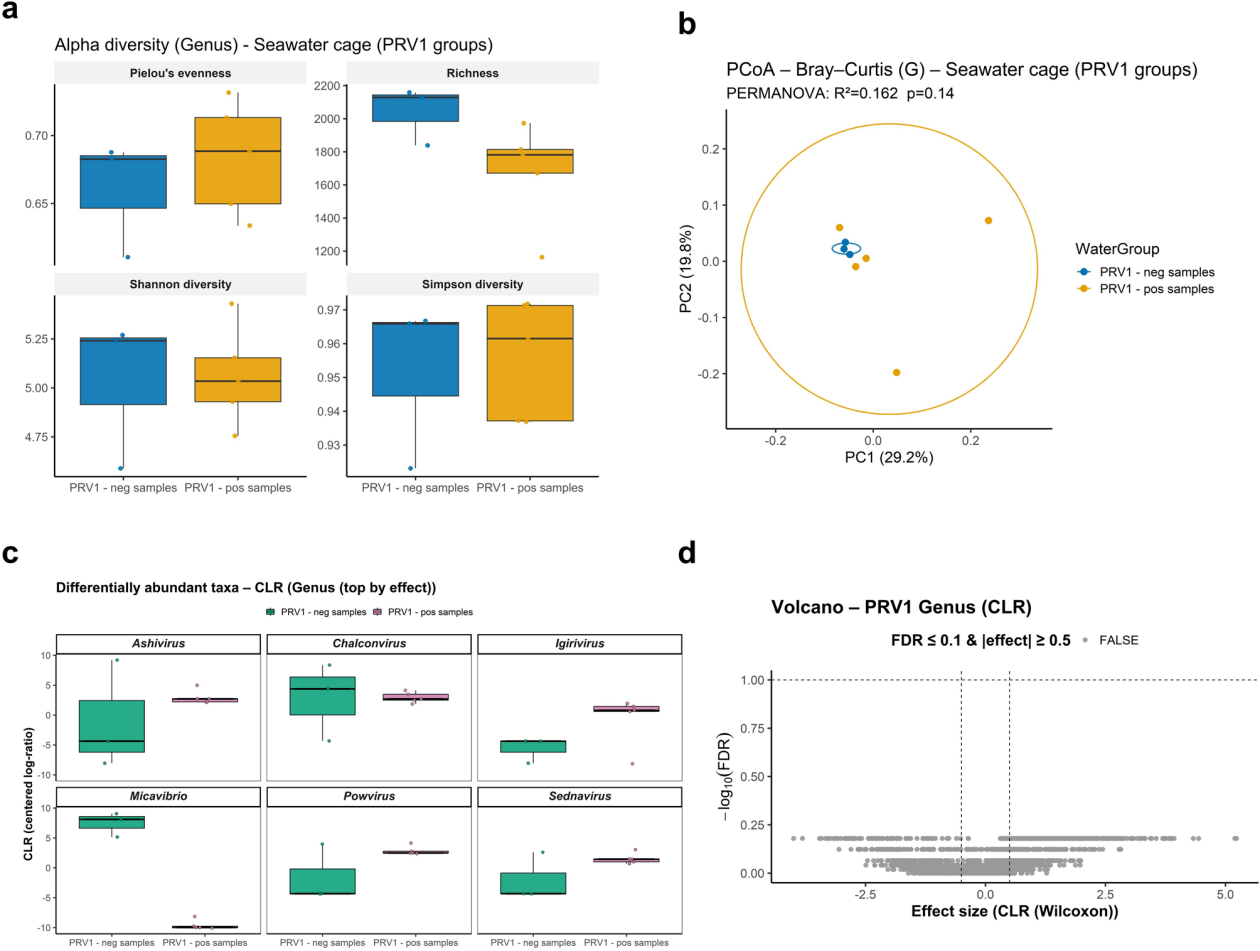
Alpha and beta diversity and differentially abundant taxa in seawater cage samples grouped by PRV1 status (positive vs. negative) at the genus level after excluding *Homo sapiens*, Archaea, and Eukaryota. (**a**) Boxplots show alpha diversity metrics (Pielou’s evenness, richness, Shannon, and Simpson) for PRV1-positive and PRV1-negative samples. (**b**) Principal Coordinates Analysis (PCoA) based on Bray–Curtis dissimilarity illustrates beta diversity patterns between PRV1 groups. PERMANOVA results (R^2^ = 0.162, *p* = 0.14) indicate no significant compositional differences. (**c**) Differential abundance analysis using CLR transformation, which highlights the top six genera with the largest effect sizes between PRV1-positive and PRV1-negative samples. (**d**) Volcano plot of CLR effect sizes (Wilcoxon test) and FDR-adjusted p-values shows no taxa meeting the significance threshold (FDR ≤ 0.1 and |effect size| ≥ 0.5).

We then analysed alpha and beta diversity within each environment, but no significant differences were observed among water sources within the same environment for any metric (Figs. 3 and 4). Since marine site samples were initially negative for PRV1 and later tested positive, we investigated whether PRV1 status (positive vs. negative) was associated with changes in alpha and beta diversity or differentially abundant taxa in seawater cage samples at the genus level. Alpha diversity did not show significant differences, although a trend toward decreased richness was observed when PRV1 was detected in water samples (Fig. 5a). Principal Coordinates Analysis (PCoA) based on Bray–Curtis dissimilarity^33^ illustrated beta diversity patterns between PRV1 groups, but PERMANOVA results (R^2^ = 0.162, *p* = 0.14) indicated no significant differences (Fig. 5b). Differential abundance analysis of the top six genera suggested a potential increase in bacteriophage-associated taxa in PRV1-positive samples (Fig. 5c), although the volcano plot showed no taxa meeting the significance threshold (FDR ≤ 0.1 and |effect size| ≥ 0.5) (Fig. 5d). The lack of taxa meeting the significance threshold likely reflects limited effect magnitudes and reduced statistical power, rather than the absence of true biological differences. This is in line with recommendations for microbiome studies, which emphasise the need to interpret effect sizes together with p-values due to the high variability and constrained sample sizes typical of environmental sequencing datasets^36^.

### Databases’ comparison

**Fig. 6:**
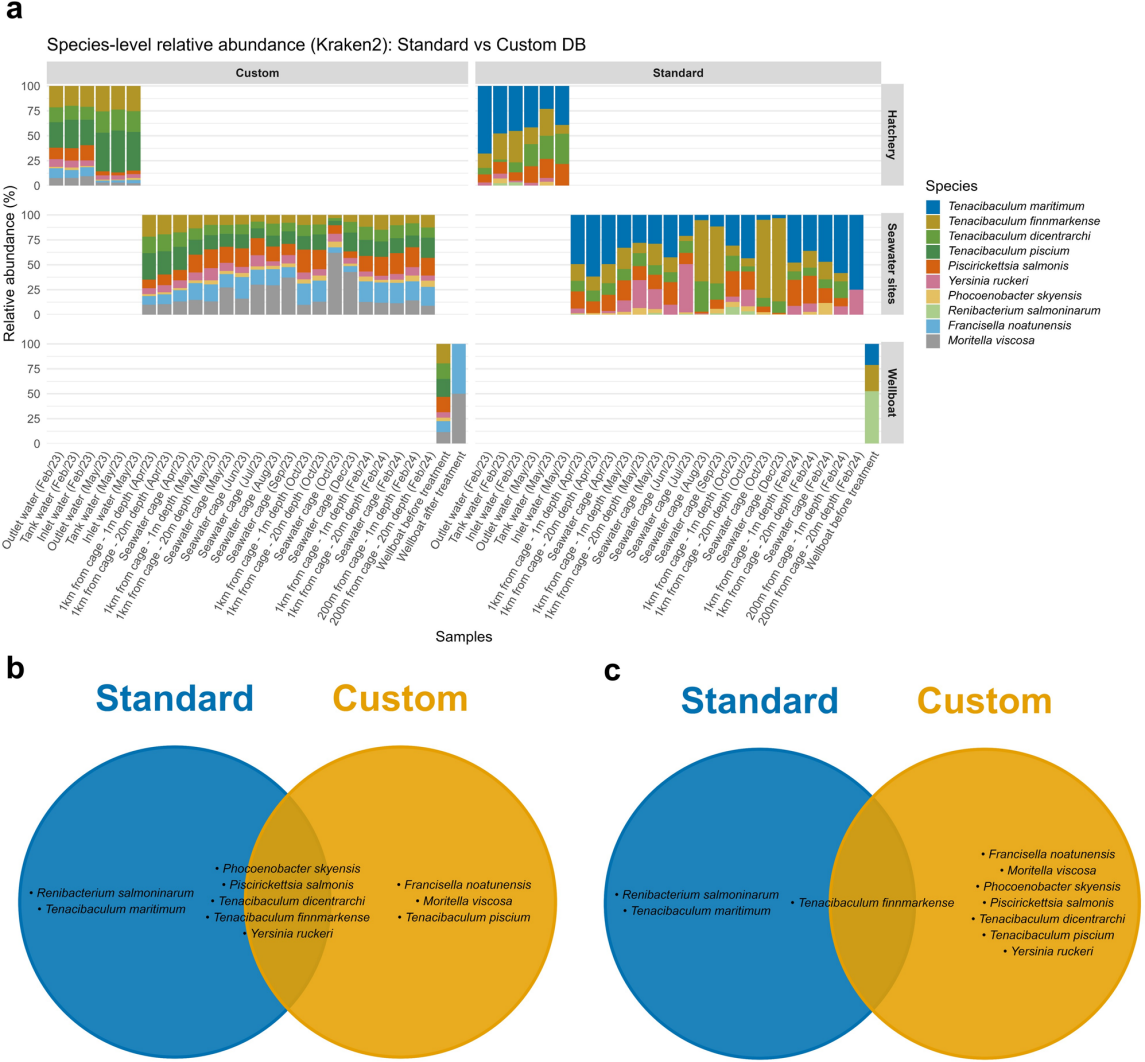
Comparison of taxonomic classification using Kraken2 with standard versus custom databases at the species level. (**a**) Relative abundance of species across samples from hatchery, seawater sites, and wellboat using the two database approaches. (**b**) Venn diagram showing species detected in hatchery and seawater site samples by the standard and custom databases. (**c**) Venn diagram showing species detected in wellboat samples by the standard and custom databases. Custom database improved detection of pathogenic species relevant to Atlantic salmon farms compared to the standard database.

We compared the taxonomic classification obtained using Kraken2 with PlusPFP vs. custom pathogen reference databases to assess their implications for environmental pathogen surveillance. Overall, the choice of database substantially influenced both detection outcomes and their interpretation. The custom pathogen reference database, which includes only well-characterised Atlantic salmon pathogens, yielded results that were more consistent with the known epidemiological context of the study sites. This comparison showed the total absence of *Francisella noatunensis*, *Moritella viscosa* and *Tenacibaculum piscium* in hatchery and seawater site samples by the PlusPFP standard database, while *Renibacterium salmoninarum* and *Tenacibaculum maritimum* were identified only from the PlusPFP standard database (Fig. 6a–c). In the wellboat water samples, the PlusPFP standard databases failed to detect most of the pathogenic species, especially after the treatment (Fig. 6a,c). These results highlight that database selection can directly influence surveillance outcomes, affecting both false positive signals and the ability to detect biologically plausible pathogens in complex environmental samples.

## Discussion

Infectious diseases remain a major challenge for the Atlantic salmon industry, and effective pathogen surveillance is essential to support timely intervention and limit pathogen spread. In this study, we evaluated the feasibility and performance of a combined qPCR–shotgun metagenomics workflow under real farming conditions, using farm-deployable filtration and portable sequencing technologies. Importantly, this work represents a single-farm, single-cohort case study in which fish remained clinically healthy throughout the production cycle and pathogen detections were consistently at low levels. This context should be considered when interpreting the results, as it limits the ability to draw strong conclusions regarding pathogen dynamics or disease-associated ecological shifts.

We used a previously established method for filtering, storing, and extracting eDNA/eRNA from water samples^18^ and tested it under real farm conditions, with the support of trained fish farm personnel who evaluated the practicality of the filtration procedure for surveillance purposes. We used specific targeted qPCR assays, and we were able to detect ISAV in all water sources (outlet-, tank- and inlet waters) at similar concentrations during the smoltification process, while PRV1 was detected in water inside the pens from August 2023. PRV1 is highly prevalent in Norwegian aquaculture: the Norwegian Fish Health Report 2024 documents that the virus was detected at 336 sites, which is a significant increase compared to numbers from 2023 (267), confirming that PRV1 circulates widely in both freshwater and seawater production environments^2^. This aligns with its widespread prevalence in production systems and wild populations. According to the farm’s fish health records, no notifiable diseases were reported throughout the full production cycle. Routine diagnostics identified low‑level ISAV (ISAV-HRP0) presence in the hatchery and PRV1 at the seawater site, but neither was associated with increased morbidity or mortality. No additional pathogens or infectious events were reported during the entire production cycle. Furthermore, the observed increase in PRV1 signal following wellboat delousing further supports the potential of water-based monitoring to capture pathogen release associated with operational stress events.

Environmental pathogen surveillance inherently involves low target concentrations, which frequently produce high Cq values near the LOD. Under these conditions, copy number estimates are subject to increased stochastic variability and should therefore be interpreted qualitatively (i.e., presence/absence) rather than quantitatively. Only detections with Cq values below this range (Cq values < 35) can be considered sufficiently robust for quantitative comparisons. Although such low-level signals should be interpreted with caution, their consistency across biological replicates and alignment with expected pathogen shedding patterns suggest that they remain informative for early detection. Notably, none of the targeted pathogens was detected outside the pens, underscoring the importance of sampling location for accurate pathogen surveillance in open-water cage systems. Hydrodynamic transport between the sea pens and surrounding waters is expected in open water aquaculture systems. Although current data for the site were not available, the low pathogen loads detected suggest that any water exchange did not obscure the differentiation between pen-adjacent and offshore samples. Future studies incorporating site-specific current measurements could help refine the spatial interpretation of waterborne pathogen signals.

We also analysed the same water samples for microbial community composition in the laboratory to assess the feasibility of using portable sequencing technologies, such as the Oxford Nanopore MinION, and to explore whether metagenomic profiles could provide complementary ecological context alongside targeted pathogen detection. The hatchery exhibited a predominance of phyla associated with nutrient-rich environments^20,21^, whereas seawater showed expected seasonal variation in microbial community composition^22,23^. Across taxonomic levels, seawater cage sites consistently displayed higher diversity and evenness, particularly at the order, family, and genus levels, compared to the hatchery environment. Richness remained similar between environments at the genus level, suggesting that observed differences were driven by community structure rather than species count. However, alpha and beta diversity showed no significant differences among water sources within the same environment. PRV1-positive samples showed subtle trends, including slightly reduced microbial richness and differences in virome composition, but these patterns were not statistically significant and should be interpreted with caution. Marine environments harbour a high abundance and diversity of bacteriophages, with community composition influenced by environmental factors such as season, temperature, photic conditions, and host availability, including taxa such as “*Candidatus* Pelagibacter” (SAR11) in cold waters^37^. Bacteriophages play a key role in marine ecosystems by regulating bacterial mortality, recycling nutrients through the viral shunt, and shaping biogeochemical cycles, particularly carbon and nitrogen fluxes^38^. In our study, *Llyrvirus*, *Pelagivirus* and *Siovirus* were more abundant in seawater sites than in the hatchery, while PRV1-positive samples showed higher abundance of *Igirivirus*, *Powvirus*, and *Sednavirus*. These viruses all belong to the *Caudoviricetes* (tailed dsDNA phages)^39^. Overall, these findings should be considered exploratory and hypothesis-generating. Given the low pathogen loads (high Cq values), the absence of clinical disease, and the potential influence of seasonal variability, the observed patterns are preliminary and cannot be interpreted as definitive evidence of dysbiosis.

Low-level human DNA contamination was detected in some samples but was taxonomically restricted and fully removed through explicit filtering of human-associated reads. Importantly, no evidence of cross-contamination with target microorganisms was observed: all negative controls were free of pathogens, and microbial profiles showed coherent ecological patterns rather than signatures of random contamination. Therefore, contamination is unlikely to have influenced the prokaryotic or viral fractions retained for downstream analysis. Although shotgun metagenomics can support antimicrobial resistance (AMR) and virulence gene profiling, this was not included here because sequencing depth and filtering parameters were optimised for taxonomic classification rather than functional annotation. In addition, the presence of human-associated contamination could confound AMR interpretation. Future studies with higher sequencing depth and stricter assembly criteria will be required to robustly integrate AMR screening into this workflow.

*Streptomyces* was detected in some samples despite no antibiotic treatments during production. Given its widespread occurrence in aquatic and soil environments, its presence likely reflects natural background microbiota rather than antimicrobial use.

No high-quality metagenome-assembled genomes (MAGs) were recovered, likely due to the limited sequencing depth achievable with portable Nanopore runs and the high complexity of marine samples. Higher coverage will be required for robust genome binning in future applications.

Despite these limitations, we established a workflow capable of extracting sufficient DNA from water samples for shotgun metagenomic sequencing, with quality metrics and positive controls confirming its robustness. Our results highlight two key considerations: (i) the persistence of human-associated contamination in field samples despite sterile precautions, and (ii) the importance of reference database selection for accurate taxonomic profiling.

While most studies rely on 16S rRNA gene sequencing^12–14^, which captures only bacterial communities, our shotgun metagenomics approach also revealed virome dynamics, underscoring the ecological role of viruses and bacteriophages in aquaculture environments. Furthermore, this workflow can be integrated with targeted DNA-based pathogen screening using curated reference databases of Atlantic salmon pathogens. Our custom database was assembled using stringent criteria, including the selection of complete or near-complete assemblies from NCBI, the exclusion of poorly annotated or fragmented genomes, and the retention only of taxa with confirmed pathogenic relevance for Atlantic salmon. The comparison between the standard Kraken2 PlusPFP database and a curated pathogen-specific reference database highlighted a key methodological consideration for environmental surveillance. In our dataset, the standard database produced apparent detections of high-consequence pathogens, such as *R. salmoninarum*, which were not supported by epidemiological context or qPCR results and are therefore likely attributable to matches to conserved genomic regions shared with environmental taxa. In contrast, the curated database reduced such ambiguous classifications and yielded results more consistent with the known health status of the fish population. This demonstrates that database choice directly influences interpretation and represents a critical step in metagenomic surveillance workflows.

This study represents a single-farm, single-cohort case conducted under clinically healthy conditions, with consistently low pathogen detections, which limits the generalizability of conclusions regarding pathogen dynamics. Within this context, our findings indicate that the integrated qPCR–metagenomics workflow is most informative under conditions of elevated pathogen activity or fish stress — for example, during disease outbreaks, following operational interventions such as delousing or grading, or when comparing multiple cohorts or production sites. Further validation across diverse farming systems, health statuses, and environmental conditions will be essential to fully assess the sensitivity, robustness, and operational value of this approach for routine farm-level decision-making.

In conclusion, this study establishes a practical baseline for continuous, water-based pathogen surveillance throughout the production cycle, combining sensitive qPCR detection with exploratory metagenomic profiling supported by curated pathogen reference databases. While shotgun metagenomics can provide additional ecological context relevant to disease risk, its application in low-biomass aquatic samples requires careful contamination control and conservative interpretation. In contrast, targeted qPCR remains a robust and sensitive tool for routine pathogen detection and represents the most reliable approach for operational surveillance in aquaculture.

## Methods

### Sampling workflow

**Fig. 7:**
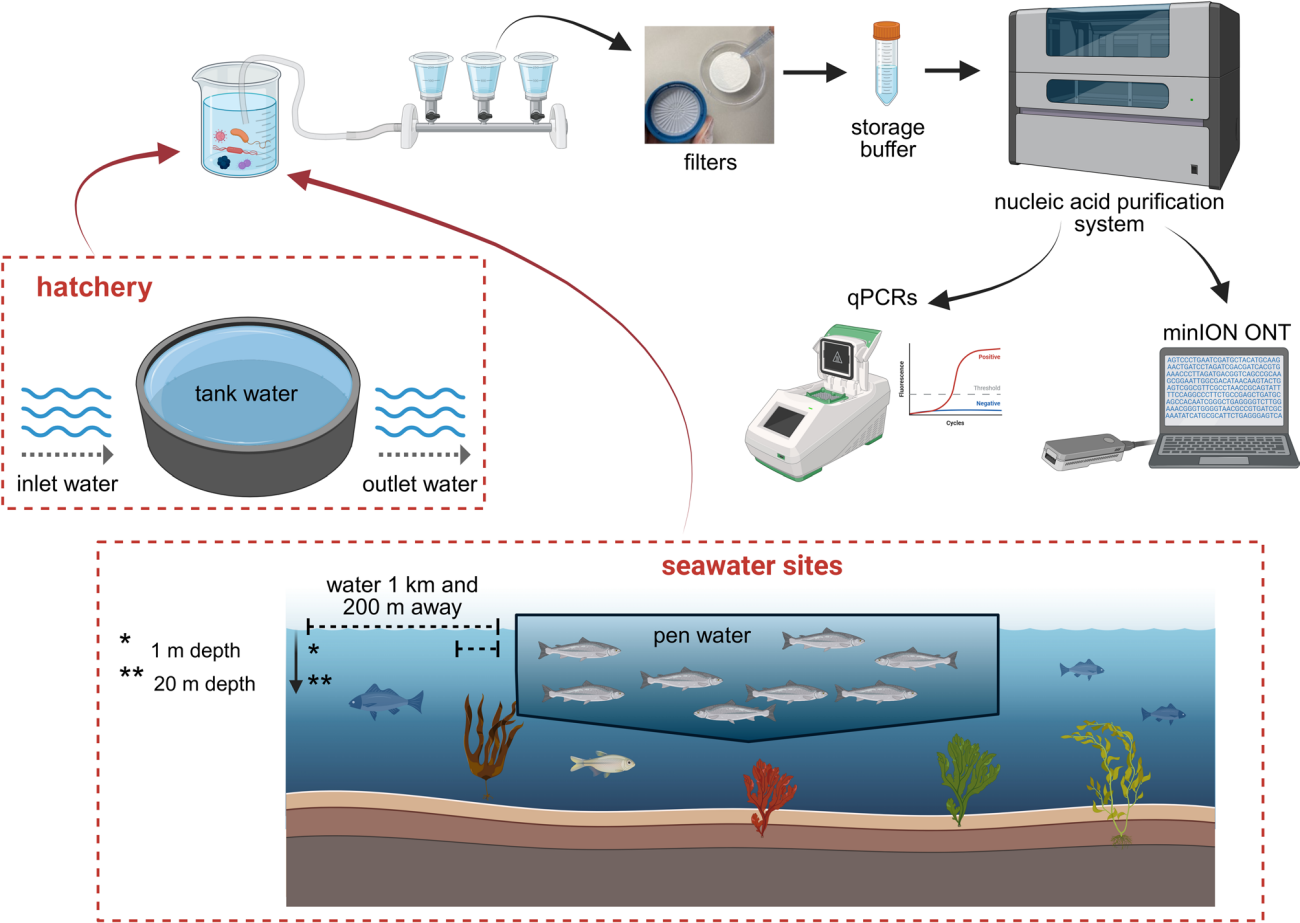
Overview of sampling workflow. Water samples were collected from two environments: hatchery (inlet, tank, and outlet water) and seawater sites (water inside the pen and locations 1 km/200m away at 1 m and 20 m depth). Samples were filtered and preserved in storage buffer before nucleic acid purification. Extracted nucleic acids were analysed by qPCR and sequenced using Oxford Nanopore Technologies (ONT) MinION platform. Created in BioRender. Benedicenti, O. (2026) https://BioRender.com/x9uc0sc.

Water samples were collected from two environments associated with the same Atlantic salmon population (ca. 250 g to 4–6 kg): a hatchery (inlet, tank, and outlet water) and a marine grow-out site. In the marine environment, samples were collected from inside the sea pen and from offshore reference sites located 1 km from the pen at 1 m and 20 m depth. During the final sampling, additional sites at 200 m were included to increase spatial resolution (Fig. 7). Hatchery samples were taken from tank outflow water, while marine samples were collected either from the dead-fish collection system (representative of pen water) or offshore by boat. For each sampling event, three biological replicates (500 mL each) were filtered on site, together with a sterile water control (autoclaved and 0.22 μm-filtered laboratory-grade water), processed using the same workflow to monitor contamination. The 500 mL volume was selected based on previous optimisation work, balancing sensitivity and filtration efficiency^18^. Larger volumes increased the risk of membrane clogging and did not improve detection probability, whereas 500 mL was sufficient to detect even low amounts of viral and bacterial pathogens in field conditions. Filtration was performed using a combined glass fibre (2.0 μm) and mixed cellulose ester membrane (0.45 μm) setup with a vacuum pump (3.8–4.0 L/min), as previously described^18^ (Fig. 7). All three biological replicates were analysed by targeted qPCR and (RT)qPCR in duplicate reactions, whereas shotgun metagenomics was performed on one pooled extract per sampling location, generated by combining the three biological replicates to ensure sufficient DNA yield for sequencing. These technical duplicates should not be interpreted as independent biological replicates, which were instead represented by the three separately collected and processed 500 mL water samples per location. This approach provided complete qPCR coverage across all environments and time points, while delivering representative metagenomic profiles for hatchery samples, all seawater sampling events, and both wellboat samples.

### eDNA/eRNA extraction and qPCR / RT-qPCR assays

Automated DNA and viral RNA extractions were performed on a MagNA Pure 96 instrument (Roche) with the MagNA Pure 96 DNA and Viral NA Large Volume Kit (Roche, France), using Pathogen Universal LV protocol with a sample input volume of 1000 µL and an elution volume of 100 µL, per sample, respectively. The pathogen panel screened at the hatchery and seawater sites was defined by the collaboration agreement with the farm. Only pathogens included in the farm’s authorised diagnostic panel could be tested, and the authors were not permitted to add additional targets. As a result, the hatchery screening did not include some freshwater-specific bacterial pathogens. This study followed the farm’s established surveillance procedures, in line with its methodological focus on evaluating a field-deployable qPCR–metagenomics workflow rather than conducting a full epidemiological assessment. In the hatchery, screening included ISAV, PRV1, piscine myocarditis virus (PMCV), infectious pancreatic necrosis virus (IPNV), and *Yersinia ruckeri*, while at marine sites the panel comprised ISAV, PRV1, PMCV, IPNV, salmonid alphavirus (SAV), and *Paramoeba perurans*.

RT-qPCR was carried out for ISAV^40^, PRV1^41^, PMCV^42^ and SAV^43^ with the Brilliant III Ultra-Fast QRT-PCR Master Mix (Agilent Technologies, USA) using the following protocol: 10 µL of 2× QRT-PCR master mix, 0.2 µL of 100 mM DTT, 1 µL of RT/RNase block, 1 µL of the 20× viral assay (6 µM of assay probe, 10 µM of each forward and reverse primers), and 5 µL extracted viral RNA. Thermocycling was performed on a CFX384 Bio-Rad and CFX-manager (software version 3.1, Bio-Rad, USA) under the following conditions: 10 min at 50 °C, 3 min at 95 °C, 45 cycles of 5 s at 95 °C and 10 s at 60 °C. Moreover, IPNV^44^ RT-qPCR was performed with the QIAGEN OneStep RT-PCR Kit (Qiagen, USA) using the following protocol: 4 µL of 5× QIAGEN OneStep RT-PCR Buffer, 0.8 µL of dNTP Mix (containing 10 mM of each dNTP), 1 µL of MgCl_2_ (25 mM), 1 µL of the 20× viral assay (6 µM of assay probe, 10 µM of each forward and reverse primers), 0.1 µL of RNaseOUT™ Recombinant Ribonuclease Inhibitor (40 U/µL, Invitrogen™, USA), 0.8 µL of QIAGEN OneStep RT-PCR Enzyme Mix, 7.3 µL of nuclease-free water, and 5 µL extracted viral RNA. Thermocycling was performed on a CFX384 Bio-Rad and CFX-manager (software version 3.1, Bio-Rad, USA) under the following conditions: 30 min at 50 °C, 15 min at 95 °C, 45 cycles of 30 s at 94 °C and 60 s at 60 °C.

qPCR was carried out for *Y. ruckeri*^45^ with TaqMan™ Fast Advanced Master Mix (Applied Biosystems™, USA) using the following protocol: 10 µL of 2× TaqMan Fast Advanced Master Mix, 5 µL of the bacterial assay (10 µM of assay probe and each forward and reverse primers), and 5 µL extracted DNA. Thermocycling was performed on a CFX384 Bio-Rad and CFX-manager (software version 3.1, Bio-Rad, USA) under the following conditions: 2 min at 50 °C, 20 s at 95 °C, 50 cycles of 3 s each at 95 °C, and 20 s at 62 °C. Specific qPCR assay for *P. perurans* was carried out with 25 µL reactions consisting of 12.5 µL TaqPath™ qPCR Master Mix, CG (Applied Biosystems™, USA), 500 nM of each primer and 250 nM of probe, nuclease-free water and 5 µL DNA sample^46^. The following qPCR cycling conditions were used: an initial denaturation at 95 °C for 20 s, followed by 50 cycles of denaturation at 95 °C for 3 s and annealing at 60 °C for 30 s^46^.

All qPCR assays were analysed in duplicate, and a no-template control (H_2_O) and water sample control from the MagNA Pure 96 extraction protocol were included on each plate as negative controls. Standard curves of serial dilution of the relevant target sequences (CFX-manager software version 3.1, Bio-Rad, USA) were used for the calculation of the concentration used for the experiments. Primers and probes are shown in the supplementary (Supplementary Table S1). Full standard curves, including amplification efficiencies, R^2^ values, slope, and y-intercept, are provided in the Supplementary Material (Table S2). These curves were generated using the same matrix composition and extraction conditions as the environmental samples and were used to calculate copy numbers and estimate assay-specific LOD.

Positive qPCR results for ISAV and PRV1 were then repeated to estimate the copy number in 5 µL with the Luna Probe One-Step RT-qPCR 4X Mix with UDG (New England Biolabs, USA) using the following protocol: 5 µL of 4× Luna Probe One-Step RT-qPCR 4X Mix with UDG, 2 µL of the viral assay (0.4 µL of 10 µM of assay probe, 0.8 µL of 10 µM of each forward and reverse primers), 5 µL extracted viral RNA, and 8 µL of nuclease-free water. Thermocycling was performed on a CFX384 Bio-Rad and CFX-manager (software version 3.1, Bio-Rad, USA) under the following conditions: 30 s at 25 °C, 10 min at 55 °C, 1 min at 95 °C, 45 cycles of 10 s at 95 °C and 30 s at 60 °C. The copy number/µL was performed using a previously designed sequence-verified synthetic gene fragments or gBlocks™ (Integrated DNA Technologies, BVBA, Leuven, Belgium)^18^. Concentrations (ng/µL) were converted to copy number/µL by using the formula provided by IDT guidelines (see Supplementary Table S2 for full calculations and dilution scheme). Serial dilutions were used to generate standard curves under the same matrix and extraction conditions as environmental samples to ensure comparable amplification efficiencies (Table S2). After optimisation, the Stock^− 4^ dilution (1.56 × 10^4^ copy number/µL) was used as a standard template to estimate the RNA copy numbers/µL for the viral pathogens (ISAV and PRV1) based on the amount captured by the filters (Table S2). To aid interpretation, qPCR copy numbers initially expressed per 5 µL of extract were converted into copies per litre of water (Table S2). Based on the standard curves generated under the same matrix and extraction conditions, the estimated limits of detection were ~ 100 copies per reaction for ISAV and ~ 10 copies per reaction for PRV1, corresponding to ~ 4 × 10^3^ copies/L and ~ 4 × 10^2^ copies/L, respectively. Several detections, particularly those with Cq > 35, fell close to these thresholds; therefore, estimates at low copy number should be interpreted cautiously due to increased stochastic variation and reduced quantification precision. Despite this uncertainty, ISAV and PRV1 detections near the LOD were consistent across biological replicates, supporting their biological relevance in the context of environmental shedding.

### DNA extraction and sequencing

For microbial community analysis, 4.5 mL of preserved pooled DNA/RNA in DNA/RNA Shield™ was extracted from each sampling point with the ZymoBIOMICS™ DNA/RNA Miniprep Kit (Zymo Research, USA, provided by Nordic Biosite AS, Sweden), following the manufacturer’s specifications. Briefly, 750 µL of liquid sample were transferred into a ZR BashingBead Lysis Tube (0.1 and 0.5 mm) and homogenised with FastPrep^®^-24 5G bead beating grinder and lysis system (MP Biomedicals, USA) using the following program: 2 cycles for 60 s (300 s of pause), speed of 6.0 m/s – Lys Matrix A, 1 mg. Samples were then centrifuged at 15,000 x g for 1 min and mixed with an equal volume (1:1) of DNA/RNA Lysis Buffer to the supernatant and processed through the DNA and RNA purification kit specifications. To elute the nucleic acids, 80 µL of nuclease-free water was added directly to the column matrix, incubated for 5 min, and then centrifuged at 15,000 x g for 2 min. The final step with the Zymo-Spin™ III-HRC Filter was also performed to remove inhibitors, salts, enzymes, or toxic compounds from the solution containing nucleic acids for sequencing purposes. Sample integrity and fragment size were assessed using the Genomic DNA (gDNA) ScreenTape assay on the TapeStation 4150 System (Agilent Technologies, USA). DNA Integrity Numbers (DIN) from water samples were greater than 6, with an average concentration of 10 ng/µL and a fragment size of approximately 10,000 base pairs. For sequencing, the Ligation Sequencing Kit V14-PCR Barcoding protocol (SQK-LSK114 with EXP-PBC001, Oxford Nanopore Technologies, UK) was applied according to the manufacturer’s instructions, with some modifications: 45 µL of each sample were used as input material, 18 cycles were performed during the PCR barcoding step, and 50 fmol were loaded onto the flow cell. Sequencing was carried out using a MinION Flow Cell (R10.4.1) on the MinION Mk1B device, with the MinKNOW™ software version 25.09.16 (https://nanoporetech.com/software/devices/minion-mk1b), excluding basecalling and saving pod5 files. Each sequencing run included two positive controls: 100 ng of the ZymoBIOMICS^®^ Microbial Community DNA Standard (Zymo Research, USA; supplied by Nordic Biosite AS, Sweden), and 45 µL of a sample from a previously published pilot experiment spiked with known concentrations of selected pathogens^18^. Negative controls were also included for each sampling point (sterile Milli-Q water filtered on site), along with an extraction control containing only DNA/RNA Shield™.

### Bioinformatic analysis

Oxford Nanopore ligation sequencing data were processed using the NEPAL pipeline (Norwegian Veterinary Institute, https://github.com/NorwegianVeterinaryInstitute/Nepal)^47^, a reproducible workflow built on Nextflow for processing raw nanopore data. The pipeline was configured to evaluate three processing strategies: simplex, duplex, and nanofilt-filtered reads, enabling comparative analysis of read quality and downstream performance. Basecalling was performed using Dorado v0.2.1 (Oxford Nanopore Technologies), integrated into the NEPAL pipeline. The super accuracy model dna_r10.4.1_e8.2_400bps_sup@v5.2.0 was used to ensure the highest possible basecalling accuracy. Nanofilt was applied to duplex reads following the filtering parameters: minimum quality score: 9; minimum read length: 300 bp (to exclude very short fragments); and maximum read length: 2,147,483,647 bp (a technical upper limit to avoid excluding long reads). Outputs from each workflow were compared in terms of read quality, length distribution, GC content, and assembly metrics. Statistical analysis among the three processing strategies was performed using the Kruskal–Wallis non-parametric test^48^ and displayed on the plots. When the Kruskal–Wallis test indicated significance (*p* ≤ 0.05), Wilcoxon rank-sum tests^34^ were performed for pairwise comparisons, and p-values were adjusted using the Benjamini–Hochberg (BH) method^35^ to control the false discovery rate. After nanofilt filtering, taxonomic classification was performed using the nf-core^49^ taxprofiler pipeline with Kraken2^50^ as the classifier. The pipeline was configured to use the Kraken2 PlusPFP database (https://benlangmead.github.io/aws-indexes/k2) and the long-read mode (--long) for optimal performance on nanopore data. To avoid host contamination, sequences from the Atlantic salmon genome (*S. salar*; reference: Ssal_v3.1; GCA_905237065.2) were excluded from the database. The pipeline generated taxonomic profiles at multiple ranks (domain, phylum, order, class, family, genus, and species) and summary reports for downstream comparative analysis. Statistical analyses and visualisation were performed in R (v4.4.0)^51^ using RStudio (v2024.04.0 + 735)^52^. After an initial taxonomic composition assessment, reads classified as *H. sapiens* and all sequences assigned to Eukaryota or Archaea were excluded to focus on prokaryotic and viral fractions relevant to fish health analyses. In the taxonomic workflow, human-associated taxa (e.g., *H. sapiens*, *Homo*, Hominidae) were explicitly removed during data processing. In the diversity workflow, the entire Eukaryota domain was filtered out in R, which also eliminated human-derived and other non-bacterial/non-viral reads. As a result, only non-host and non-human microbial taxa were retained for all taxonomic, alpha-diversity, beta-diversity, and differential abundance analyses. Alpha diversity indices—species richness, Shannon diversity, Simpson diversity, and Pielou’s evenness—were calculated from relative abundances. Group comparisons (e.g., hatchery vs. seawater sites or water sources in each environment) used Wilcoxon rank-sum^34^ or Kruskal–Wallis^48^ tests with FDR correction (Benjamini–Hochberg^35)^. Beta diversity was based on Bray–Curtis dissimilarity^33^ and visualised with PCoA. Group separation was assessed using PERMANOVA (*adonis2*^53^) and multivariate dispersion tests (*betadisper*^54)^, as implemented in the vegan R package^55^. Confidence ellipses (95%) were drawn for visual separation among environments. Differential abundance analyses were assessed with centred log-ratio (CLR) transformation and Dirichlet-Monte-Carlo model using the *ALDEx2* package^56^, a negative binomial model to raw counts was used to estimate log₂ fold changes between groups with the *DESeq2* package^57^, and non-parametric comparison of log-ratio-transformed relative abundances were assessed with Wilcoxon rank-sum^34^ test with FDR correction (Benjamini–Hochberg^35^(FDR ≤ 0.10 and |effect size| ≥ 0.5). We applied an FDR threshold of ≤ 0.10, consistent with recommendations for flexible false discovery control in large-scale omics analyses, where rigid cut-offs (e.g., FDR ≤ 0.05) may be overly conservative and reduce sensitivity in high-dimensional datasets^58^. We report effect sizes alongside p-values, following recommendations that highlight the importance of effect magnitude in interpreting microbiome analyses, particularly in studies where statistical power may be limited^36^. A Custom Kraken2 database was developed to compare the taxonomic classification of fish pathogens with the standard Kraken2 PlusPFP database. The Standard PlusPFP database includes bacterial, archaeal, viral, fungal, and protozoan genomes distributed with Kraken2. The Custom database was built with reference genomes and complete assemblies of known Atlantic salmon pathogens, including *R. salmoninarum*, Salmon gill poxvirus, *Phocoenobacter skyensis*, *Phocoenobacter atlanticus* subsp. *atlanticus*, *Y. ruckeri*, *M. viscosa*, *Aeromonas salmonicida* subsp. *salmonicida*, *Piscirickettsia salmonis*, *T. maritimum*, *Tenacibaculum dicentrarchi*, *T. piscium*, *Tenacibaculum finnmarkense*, and *F. noatunensis*. Genomes were retrieved from NCBI RefSeq/GenBank, and they were manually curated to ensure completeness and taxonomic accuracy. The new entries were added to the Kraken2 library, and the database index was rebuilt. Comparative visualisations (species-level relative abundance barplots and Venn overlaps) were generated in R to identify taxa uniquely detected by either database.

## Supplementary Information

Below is the link to the electronic supplementary material.

Supplementary Material 1

Supplementary Material 2

Supplementary Material 3

Supplementary Material 4

Supplementary Material 5

Supplementary Material 6

Supplementary Material 7

## Data Availability

The datasets generated during and/or analysed during the current study are available at the BioProject PRJNA1379277 (https://www.ncbi.nlm.nih.gov/bioproject/PRJNA1379277). All other data generated during this study are included in this published article and its Supplementary Information files.
