# Supplementary material for: Integrated approaches for pathogen monitoring and shotgun metagenomic analysis in Atlantic salmon farming
Source: Sci Rep. 2026 Apr 14;16:17468. doi: 10.1038/s41598-026-48791-x (PMC13237240; doi:10.1038/s41598-026-48791-x)
Supplement: Supplementary file 1 — Supplementary Material 1 [file 41598_2026_48791_MOESM1_ESM.docx]

**Table S1. Primer and probe sequences used for qPCR assays.** All assays listed have undergone recent analytical verification within the Norwegian Veterinary Institute diagnostic pipeline to ensure detection of currently circulating pathogen variants. The ISAV and SAV assays correspond to the WOAH‑ and EU‑RL‑recommended diagnostic methods targeting conserved genomic regions.

| **Pathogen** | **Oligonucleotides and probe** |
| --- | --- |
| ISAV^1^ | FW: 5’- CTACACAGCAGGATGCAGATGT -3’  REV: 5’- CAGGATGCCGGAAGTCGAT -3’  Probe: 5’-6-FAM- CATCGTCGCTGCAGTTC -MGB-NFQ-3’ |
| PRV1^2^ | FW: 5’- TGCTAACACTCCAGGAGTCATTG -3’  REV: 5’- TGAATCCGCTGCAGATGAGTA -3’  Probe: 5’-6-FAM- CGCCGGTAGCTCT -MGB-NFQ-3’ |
| SAV^3^ | FW: 5’- CCGGCCCTGAACCAGTT -3’  REV: 5’- GTAGCCAAGTGGGAGAAAGCT -3’  Probe: 5’-6-FAM- CTGGCCACCACTTCGA -MGB-NFQ-3’ |
| PMCV^4^ | FW: 5’- TTCCAAACAATTCGAGAAGCG -3’  REV: 5’- ACCTGCCATTTTCCCCTCTT -3’  Probe: 5’-6-FAM - CCGGGTAAAGTATTTGCGTC -MGB-NFQ-3’ |
| IPNV^5^ | FW: 5’- CGACCGACATGAACAAAATCA -3’  REV: 5’- AGTTGCAGCTGTATTCGCACA -3’  Probe: 5’-6-FAM - TCTAGCCAACAGTGTGTACGGCCTCCC - BHQ-3’ |
| *Yersinia ruckeri* CC1^6^ | FW: 5’- GAATTAGGCGCAACTCAATTTGAC -3’  REV: 5’- ACTGGTAAGGGATGTTATGTTTCA -3’  Probe: 5’-VIC- TATGACGACTGAGTGTTTAC -MGB-NFQ-3’ |
| *Paramoeba perurans*^7^ | FW: 5’- GTTCTTTCGGGAGCTGGGAG -3’  REV: 5’- CATGATTCACCATATGTTAAATTTCC -3’  Probe: 5’-6-FAM - CTCCGAAAA/ZEN/GAATGGCATTGGCTTTTGA -3IABkFQ-3’ |


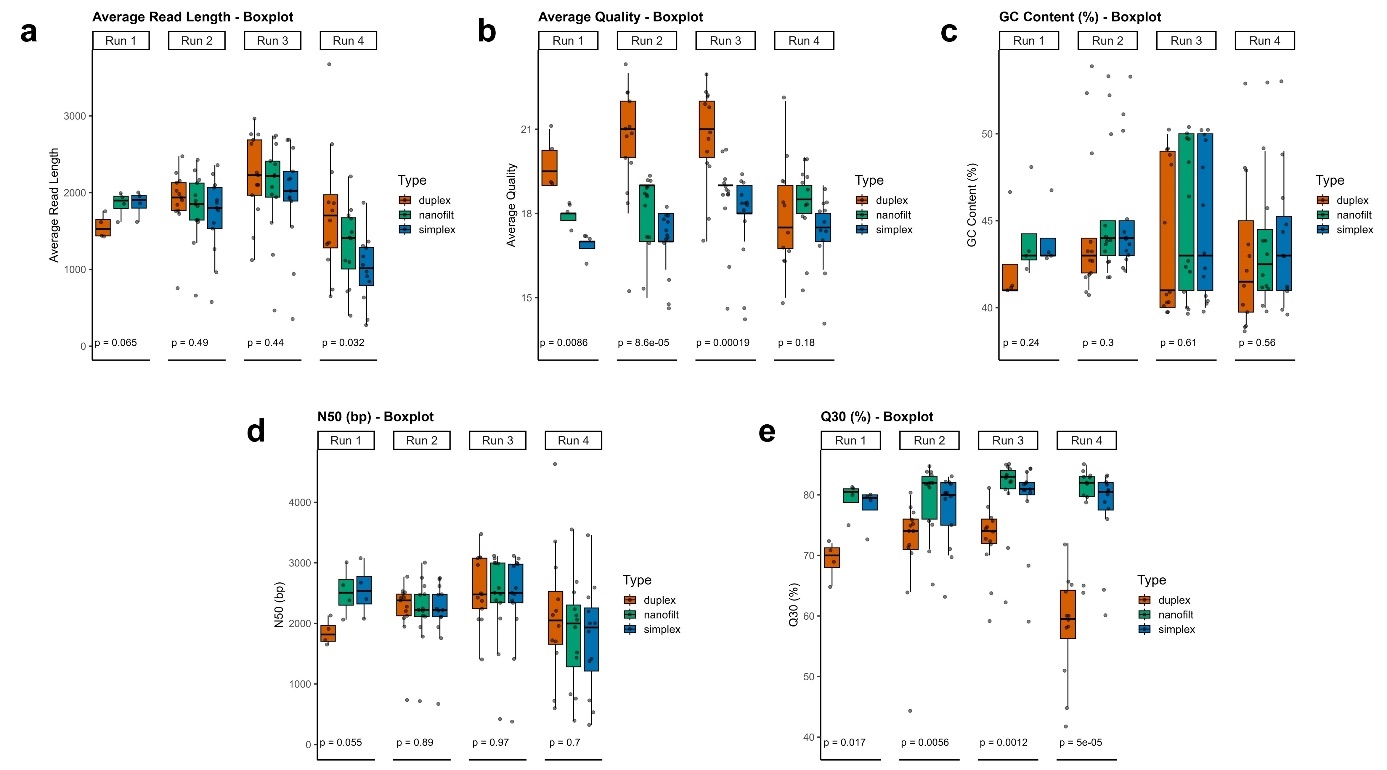


**Fig. S1. Comparative boxplots showing sequencing performance metrics across four experimental runs for three workflow pipeline analyses using the Nepal pipeline.** Distributions of five sequencing metrics are presented for runs 1–4, with methods distinguished by colour: duplex (orange), nanofilt (green), and simplex (blue). **(a)** Average Read Length represents the mean sequence length per read, **(b)** Average Quality is the mean base quality score, **(c)** GC Content (%) is the percentage of GC bases in reads, **(d)** N50 (bp) is the N50 value in base pairs, indicating read length distribution, and **(e)** Q30 (%) is the percentage of bases with quality score ≥ Q30. Kruskal–Wallis test p-values are shown below each plot, indicating overall differences among workflows for each run.


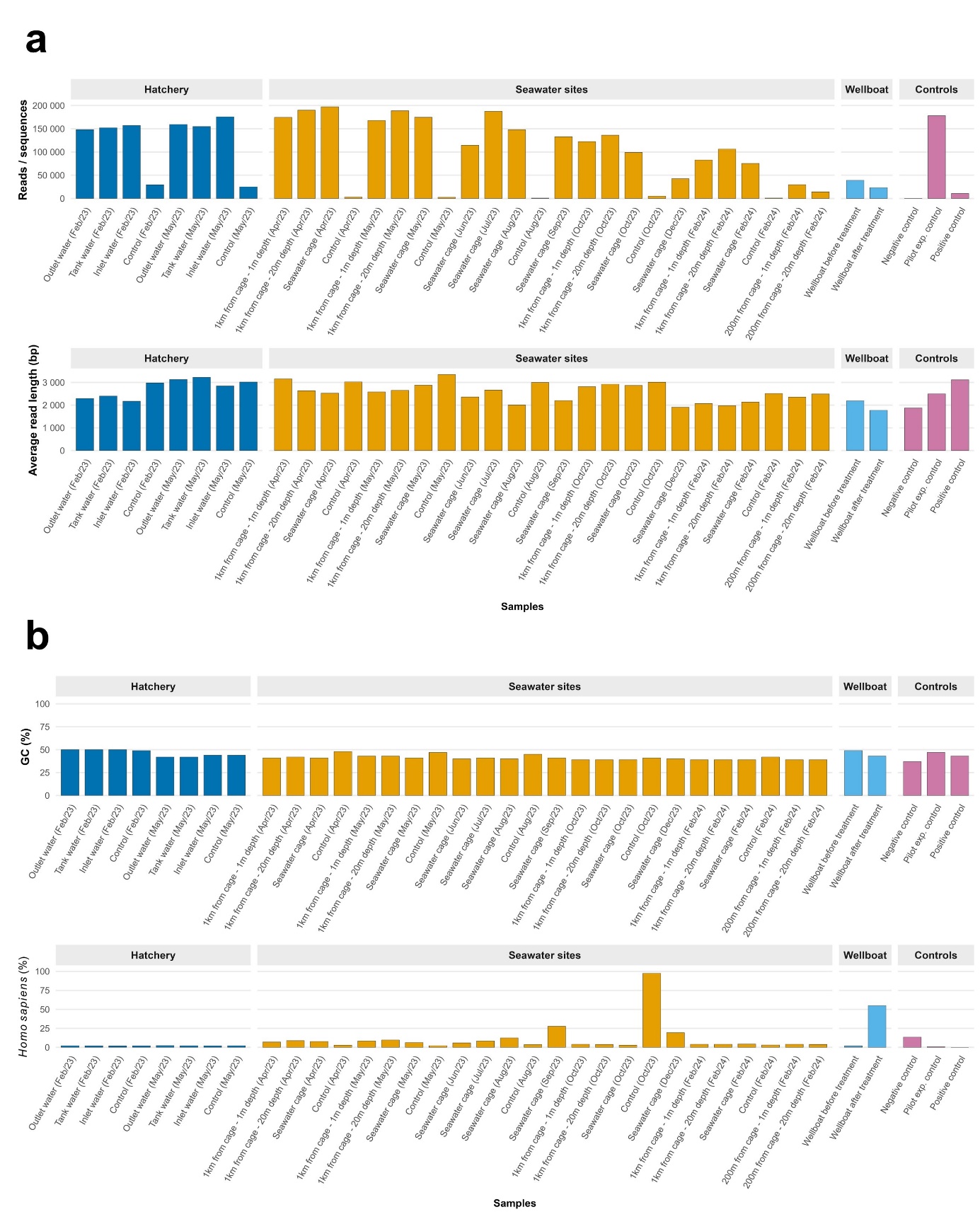


**Fig. S2. Bar plots display per-sample sequencing metrics and the proportion of human reads across the Hatchery, Seawater, Wellboat, and Control groups using the nf-core taxprofiler pipeline.** **(a)** Reads / sequences on the top represent the total number of reads after quality-trimming (from MultiQC “post-trimming total sequences”) and the average read length (bp) on the bottom is the mean read length after trimming; **(b)** GC (%) on the top is the average GC content of processed reads and Homo sapiens (%) on the bottom shows the proportion of classified reads assigned to H. sapiens relative to all classified reads (from Kraken2 Top-N report). Metrics were extracted from MultiQC and Kraken2 outputs and visualised in R classification results using a custom R script (R v4.4.0, packages tidyverse, ggplot2, cowplot).


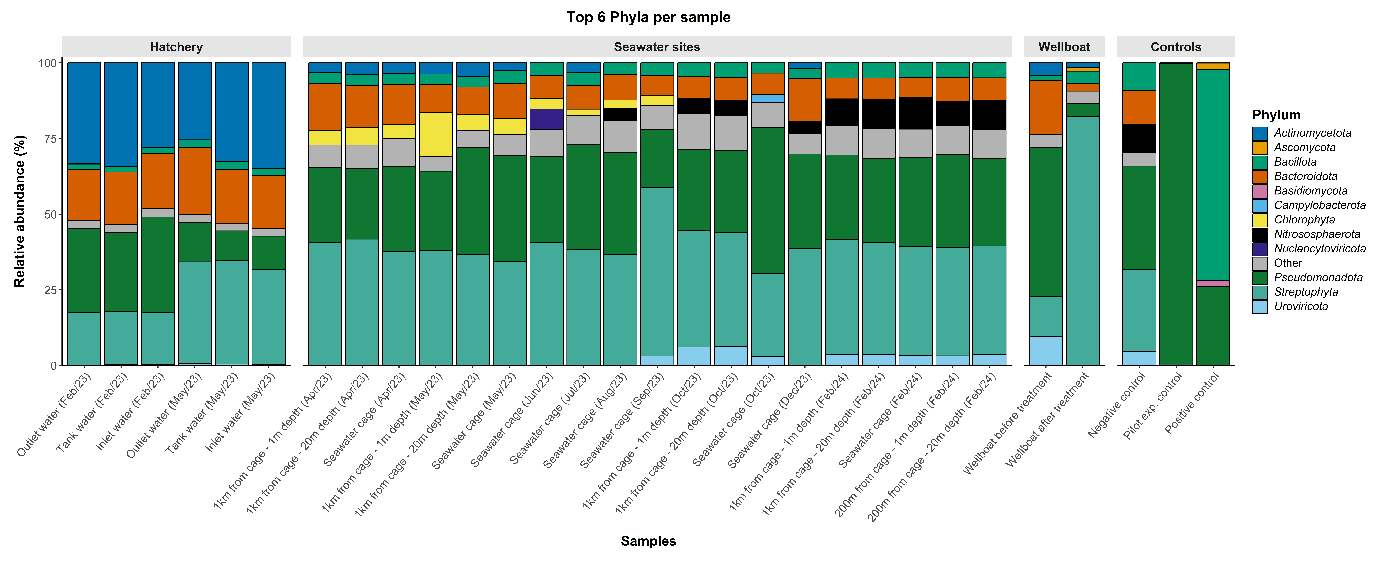


**Fig. S3.** **Relative abundance (%) among the top 6 classified phyla.** Bar plots display relative abundances (%) of the top 6 phyla per sample and all other phyla grouped as “Other”. Samples are grouped by environment (Hatchery, Seawater sites, and Wellboat) and controls. Post-nanofilt Kraken2 taxonomic profiles were calculated without host and contaminant reads (i.e., Salmo salar and H. sapiens), and unclassified reads were not included in relative abundance but calculated separately for each sample.

**References**

1 Snow, M. *et al.* Development, application and validation of a Taqman real-time RT-PCR assay for the detection of infectious salmon anaemia virus (ISAV) in Atlantic salmon (*Salmo salar*). *Developments in Biologicals* **126**, 133-145; discussion 325-136 (2006).

2 Palacios, G. *et al.* Heart and Skeletal Muscle Inflammation of Farmed Salmon Is Associated with Infection with a Novel Reovirus. *PLOS One* **5** (2010). <https://doi.org:https://doi.org/10.1371/journal.pone.0011487>

3 Hodneland, K. & Endresen, C. Sensitive and specific detection of Salmonid alphavirus using real-time PCR (TaqMan®). *J Virol Methods* **131**, 184-192 (2006). <https://doi.org:10.1016/j.jviromet.2005.08.012>

4 Løvoll, M. *et al.* A novel totivirus and piscine reovirus (PRV) in Atlantic salmon (*Salmo salar*) with cardiomyopathy syndrome (CMS). *Virol J* **7**, 309 (2010). <https://doi.org:https://doi.org/10.1186/1743-422X-7-309>

5 Ørpetveit, I. *et al.* Detection of infectious pancreatic necrosis virus in subclinically infected Atlantic salmon by virus isolation in cell culture or real-time reverse transcription polymerase chain reaction: influence of sample preservation and storage. *Journal of Veterinary Diagnostic Investigation* **22**, 886-895 (2010). <https://doi.org:https://doi.org/10.1177/10406387100220060>

6 Riborg, A. *et al.* qPCR screening for *Yersinia ruckeri* clonal complex 1 against a background of putatively avirulent strains in Norwegian aquaculture. *Journal of Fish Diseases* **45**, 1211-1224 (2022). <https://doi.org:10.1111/jfd.13656>

7 Lazado, C. C., Breiland, M. W., Furtado, F., Burgerhout, E. & Strand, D. The circulating plasma metabolome of *Neoparamoeba perurans*-infected Atlantic salmon (*Salmo salar*). *Microbial Pathogenesis* **166**, 105553 (2022). <https://doi.org:https://doi.org/10.1016/j.micpath.2022.105553>
